# Supplementary material for: Methylation clocks fail to generalize across genetically admixed individuals
Source: eLife. 2026 Aug 4;14:RP105343. doi: 10.7554/eLife.105343 (PMC13436966; doi:10.7554/eLife.105343)
Supplement: Peer review history

# Editor report: eLife Assessment

**DOI:** 10.7554/eLife.105343.3.sa0

**Authors:** Jenny Tung

**Affiliations:** Max Planck Institute for Evolutionary Anthropology Germany

**Keywords:** Convincing, Important

This **important** study assesses the portability of epigenetic clocks across ancestries, including in the context of accelerated aging in Alzheimer's Disease patients. It provides **convincing** evidence for population differences in age estimation accuracy across a variety of epigenetic clocks, driven in large part by continuous variation in ancestry. Given the accelerating use of epigenetic clocks across fields, this study is likely to be of interest to researchers working on human genetic and epigenetic variation or who apply epigenetic clocks to diverse human populations.

# Referee report: Reviewer #3 (Public review)

**DOI:** 10.7554/eLife.105343.3.sa1

The authors find that DNA methylation-based clocks are generally less accurate at predicting age in cohorts with large proportions of non-European (especially African) ancestry, compared to cohorts with high European ancestry proportions (which more closely reflects the genetic composition of individuals included in training sets). They provide evidence for this ancestry bias via ancestry-stratified analyses, and in analyses of continuous ancestry proportion effects on clock error. They then test two hypothesized underlying causes of ancestry bias: that ancestry-differentiated SNPs disrupt CpG sites preventing methylation, and that ancestry-differentiated SNPs influence DNA methylation levels. They find clear evidence especially for the second cause, in the form of meQTL that influence clock CpG sites and vary in frequency across ancestry groups. Finally, the authors provide key discussions of potential paths forward to alleviate bias and improve portability for future clock algorithms.

The topic is timely due to the increasing popularity of DNA methylation-based clocks and the acknowledgment that many algorithms (e.g., polygenic risk scores) lack portability when applied to cohorts that substantially differ in ancestry or other characteristics from the training set. This has been discussed to some degree for DNA methylation-based clocks, but could of course use more discussion and empirical attention, which the authors nicely provide using an impressive and diverse collection of data. The inclusion of data from multiple cohorts, the analysis of ancestry as a continuous variable, and the attempts to address the underlying causes of ancestry-based differences in accuracy provide comprehensive evidence that genetic background influences clock portability.

# Author comment: Author response

**DOI:** 10.7554/eLife.105343.3.sa2

**Authors:** Sebastián Cruz-Gonzalez, Ogechukwu Okpala, Esther Gu, Lissette Gomez, Makaela Mews, Jeffery M Vance, Michael L Cuccaro, Mario R Cornejo-Olivas, Briseida E Feliciano-Astacio, Goldie S Byrd, Jonathan Haines, Margaret A Pericak-Vance, Anthony J Griswold, William S Bush, John A Capra

**Affiliations:** Biological and Medical Informatics Program, University of California, San Francisco San Francisco United States; University of California, San Francisco San Francisco United States; University of Miami Miami United States; University of Miami Miami United States; Case Western Reserve University Cleveland United States; University of Miami Miami United States; University of Miami Miami United States; Instituto Nacional de Ciencias Neurologicas Lima Peru; Universidad Central del Caribe Bayamon Puerto Rico; Wake Forest University Winston-Salem United States; Case Western Reserve University Cleveland United States; University of Miami Miami United States; University of Miami Miami United States; Case Western Reserve University Cleveland United States; University of California, San Francisco San Francisco United States

The following is the authors’ response to the original reviews.

**Reviewer #1 (Public review):**

Summary:

Cruz-Gonz´alez and colleagues draw on DNA methylation and paired genetic data from 621 participants (n=308 controls; n=313 participants with Alzheimer’s Disease). The authors generate a panel of epigenetic biomarkers of aging with a primary focus on the Horvath multi-tissue clock. The authors find weaker correlations between predicted epigenetic age and chronological age in subgroups with higher African ancestry than within a subgroup identified as White. The authors then examine genetic variation as a potential source for between-group differences in epigenetic clock performance. The authors draw on a large collection of publicly available methylation quantitative trait loci datasets and find evidence for substantial overlap between clock CpGs located within the Horvath clock and methQTLs. Going further, the authors show that methQTLs that overlap with Horvath clock CpGs show greater allelic variation in African ancestral groups pointing to a potential explanation for poorer clock performance within this group.

Thank you for this summary.

Strengths:

This is an interesting dataset and an important research question. The authors cite issues of portability regarding polygenic risk scores as a motivation to examine between-group differences in the performance of a panel of epigenetic clocks. The authors benefit from a diverse cohort of individuals with paired genetic data and focus on a clinical phenotype, Alzheimer’s disease, of clear relevance for studies evaluating age-related biomarkers.

Thank you.

Weaknesses:

While the authors tackle an important question using a diverse cohort the current manuscript is lacking some detail that may diminish the potential impact of this paper. For example:

(1) Information on chronological ages across groups should be reported to ensure there are no systematic differences in ages or age ranges between groups (see point below).

Thank you for pointing out this omission. The distributions are now presented in Supplementary Figure 1. While there is some variation in median age, the age ranges are similar across cohorts (median 73.1 to 79.3). The small differences do not explain the differences in accuracy between the cohorts, e.g., the median age of the African Americans (76.4) is lower than the median age for the White cohort (77.7).

(2) The authors compare correlations between chronological age and epigenetic age in sub-groups within to correlations reported by Horvath (2013). Attempting to draw comparisons between these two datasets is problematic. The current study has a much smaller N (particularly for sub-group analyses) and has a more restricted age range (60-90yrs versus 0-100 yrs). Thus, is an alternative explanation simply that any weaker correlations observed in this study are driven by sample size and a restricted age range? Reporting the chronological ages (and ranges) across subgroups in the current study would help in this regard. Similarly, given the lack of association between AD status and epigenetic age (and very small effect in the white group), it may be of interest to examine the correlation between chronological age and epigenetic age in each group including the AD participants: would the between-group differences in correlations between chronological age and epigenetic be altered by increasing the sample size?

Our conclusions about the reduced accuracy of the clock in admixed individuals are based on the comparison within the MAGENTA cohorts, not a comparison of MAGENTA to previously published studies. We find significantly reduced accuracy in the admixed cohorts compared to the White MAGENTA cohort. Further supporting this conclusion beyond he MAGENTA cohort, we analyzed three independent whole blood methylation datasets. Two focused on African American individuals—the Grady Trauma Project (n = 422) and the GENOA study (n = 1,394)—and one focused on White Swedish individuals (n = 729). As observed in MAGENTA, the Horvath clock had significantly lower accuracy for the African American cohorts Figure 3 than for the White Swedish cohort.

When comparing results across studies, the reviewer is correct that lower correlations are generally seen for older cohorts. Indeed, other studies applying the Horvath clock have seen similar correlations in older cohorts to those observed in MAGENTA (Marioni et al., 2015, Horvath 2013, and Shireby et al., 2020). We now also include the chronological age distributions of the cohorts in this study, along with their mean and standard deviations (Supplementary Figure 1). This shows that the distribution of chronological ages for White individuals is similar to the cohorts where the clocks did not perform as well. Finally, as suggested, we correlated chronological and epigenetic age with the inclusion of AD cases in each cohort for the Horvath clock. The significantly lower performance of the clock on Puerto Ricans and African Americans, relative to White individuals, remains even after including all individuals in each cohort. Thus, combining cases and controls did not qualitatively change the performance relationships for the African Americans and Puerto Ricans relative to the Whites (Supplementary Figure 3).

(3) The correlation between chronological age and epigenetic age, while helpful is not the most informative estimate of accuracy. Median absolute error (and an analysis of MAE across subgroups) would be a helpful addition.

We used correlation because it is commonly used to evaluate the performance of epigenetic age clocks, but we agree that other error quantification metrics provide a complementary perspective. We now include MAE and MSE comparisons across sub-groups in the revision (Supplementary Table 1). We find that across all accuracy metrics, the African American and Puerto Rican cohorts perform worse than the White and Peruvian cohorts. Interestingly, the Cubans show relatively high error despite a high correlation between predicted and chronological age. However, there are only 21 non-demented Cuban controls. In addition, we evaluated the same metrics in three replicate datasets (two African American cohorts and one for White Swedish individuals) and found the same patterns of lower accuracy across metrics in African ancestry individuals, albeit with some variation in accuracy between cohorts (Supplementary Table 2). Notably, as discussed above, this is not driven by differences in chronological age distributions: when we subset to older individuals (≥ 55 years old) in order to facilitate comparisons to MAGENTA study individuals, the median age for the White Swedish individuals (70 years old) is higher than that of the GENOA (62.7 years old) and Grady (58 years old) individuals. Despite the difference in median ages, the clock performs better on White Swedish individuals across all accuracy metrics than the African ancestry cohorts with younger individuals.

(4) More information should be provided about how DNAm data were generated. Were samples from each ancestral group randomized across plates/slides to ensure ancestry and batch are not associated? How were batch effects considered? Given the relatively small sample sizes, it would be important to consider the impact of technical variation on measures of epigenetic age used in the current study. The use of principal Component-based versions of these clocks (Higgins Chen et al., 2023; Nature Aging https://doi.org/10.1038/s43587-022-00248-2) may help address concerns such concerns.

Thank you for pointing out the need for additional context on data generation. We have added details to the Methods. All omics data from the MAGENTA study were generated using standard protocols that ensure minimal technical artifacts and batch effects. Samples were randomized across plates and chips to ensure that ancestry, age, and sex were not confounded with each batch. We also performed a principal components analysis of the normalized methylation data used as inputs for all MAGENTA analyses. We found that the samples did not stratify by sample plate, cohort, ethnicity, or ascertainment center along the principal components (Supplementary Figure 2).

We also thank the reviewer for their suggestion to apply the principal component clock to account for potential technical variation. As outlined in the new section “Principal component versions of the methylation clocks also have lower age prediction accuracy for genetically admixed individuals,” using the principal component version of the Horvath clock did not result in consistent improvement in age prediction accuracy or generalization across MAGENTA cohorts (Supplementary Figures 4 and 5). The lower accuracy for age prediction in individuals with substantial African ancestry was present for the PC clock in the replication cohorts, just as in the MAGENTA cohorts (Supplementary Figure 6).

(5) Marioni et al., (2015) found a very weak cross-sectional association between DNAm Age and cognitive function (r∼0.07) in a cohort of >900 participants. Given these effect sizes, I would not interpret the absence of an effect in the current study to reflect issues of portability of epigenetic biomarkers.

We agree that previous links between DNAm Age and AD or cognitive function have been relatively small in magnitude. For example, the PhenoAge paper (Levine et al., 2018) and a study using the Horvath clock (Levine et al., 2015) found age acceleration of less than a year in AD patients relative to non-demented individuals. Similar results have also been observed in studies with smaller sample sizes (e.g., 700 for Levine et al. 2015 and 604 for Levine et al. 2018). Given these small effect sizes, we agree that accounting for statistical power is essential for interpretation of our results. We performed power calculations based on an effect of the size observed in previous studies (0.5 year acceleration). We have 86% power in the full MAGENTA data set to detect an effect of this size. Stratifying by cohorts, we have 75% power for the African Americans, 72% for the Puerto Ricans, 72% for the Whites, 65% for the Peruvians, and 47% for the Cubans. Thus, we believe we have high enough power that the consistent lack of association outside of the White cohort in MAGENTA is likely meaningful. Based on these calculations, there is only a 1% chance that we would not observe an effect in any of the other cohorts if the effect was present across cohorts. Nonetheless, we have added caveats about power and the small sample size to our suggestion that the reduced accuracy of the clocks contributes to the lack of AD association outside of Whites.

(6) The methQTL analyses presented are suggestive of potential genetic influence on DNAm at some Horvath CpGs. Do authors see differences in DNAm across ancestral groups at these potentially affected CpGs? This seems to be a missing piece together (e.g., estimating the likely impact of methQTL on clock CpG DNAm).

We agree. Thank you for this suggestion. We have added Figure 6 in the main text to address this gap. In short, we analyzed additional whole blood methylation data from inidividuals with African ancestry and found that a substantial proportion of the CpGs in methylation clocks are differentially methylated in African ancestry individuals relative to European ancestry individuals. In the case of the Horvath clock, we find that 84/353 (23.8%) of the clock CpGs are differentially methylated between ancestries. In parallel, we found that 56 of these differentially methylated clock CpGs are also affected by meQTL, many of which are at different frequencies between populations. We also investigated whether the meQTL-affected clock CpGs are associated with increased clock error in the MAGENTA individuals. We found 56 clock CpGs whose methylation levels associated with increased clock error, and 42 of these have at least one meQTL. Thus, while meQTL are not the only factor to affect the portability of methylation clocks across global populations, we suggest that they are a significant contributor, especially in the case of the Horvath clock.

**Reviewer #2 (Public review):**

Summary:

This paper seeks to characterize the portability of methylation clocks across groups. Methylation clocks are trained to predict biological aging from DNA methylation but have largely been developed in datasets of individuals with primarily European ancestries. Given that genetic variation can influence DNA methylation, the authors hypothesize that methylation clocks might have reduced accuracy in non-European ancestries.

Strengths:

The authors evaluate five methylation clocks in 621 individuals from the MAGENTA study. This includes approximately 280 individuals sampled in Puerto Rico, Cuba, and Peru, as well as approximately 200 self-identified African American individuals sampled in the US. To understand how methylation clock accuracy varies with proportion of non-European ancestry, the authors inferred local ancestry for the Puerto Rican, Cuban, Peruvian, and African American cohorts. Overall, this paper presents solid evidence that methylation clocks have reduced accuracy in individuals with non-European ancestries, relative to individuals with primarily European ancestries. This should be of great interest to those researchers who seek to use methylation clocks as predictors of age-related, late-onset diseases and other health outcomes.

Thank you for this summary.

Weaknesses:

One clear strength of this paper is the ability to do more sophisticated analyses using the local ancestry calls for the MAGENTA study. It would be valuable to capitalize on this strength and assess portability across the genetic ancestry spectrum, as was recently advocated by Ding et al. in Nature (2023). For example, the authors could regress non-European local ancestry fraction on measures of prediction accuracy. This could paint a clearer picture of the relationship between genetic ancestry and clock accuracy, compared to looking at overall correlations within each cohort.

Thank you for this suggestion. To model portability across genetic ancestry as a spectrum, we regressed the Horvath clock error on the proportions of African ancestry in the genomes of the MAGENTA individuals, adjusting for chronological age. The proportion of African ancestry is significantly associated with increased Horvath clock error (p = 0.039), with the clock making less accurate age predictions by 1.46 years for individuals with full African ancestry compared to no African ancestry. We have added this new analysis to the Results.

The authors present two possible reasons that methylation clocks might have reduced accuracy in individuals with non-European ancestries: genetic variants disrupting methylation sites (i.e., ”disruptive variants”) and genetic variants influencing methylation sites (i.e., meQTLs). The authors conclude disruptive variants do not contribute to poor methylation clock portability, but the evidence in support of this conclusion is incomplete. The site frequency spectrum of disruptive variants in Figure 4 is estimated from all gnomAD individuals, and gnomAD is comprised of primarily European individuals. Thus, the observation that disruptive variants are generally rare in gnomAD does not rule them out as a source of poor clock portability in admixed individuals with non-European ancestries.

In the revision, we now additionally report ancestry-specific allele frequencies to demonstrate the rarity of CpGclock disrupting variants (Supplementary Figure 9). The global allele frequencies were so low that even if they all occurred in individuals of non-European ancestries, they would still be extremely rare.

It is also unclear to what extent meQTLs impact methylation clock portability. The authors find that the frequency of meQTLs is higher in African ancestry populations, but this could reflect the fact that some of the analyzed meQTLs were ascertained in African Americans. The number of meQTL-affected methylation sites also varies widely between clocks, ranging from 6 to 271; thus, meQTLs likely impact the portability of different clocks in different ways. Overall, the paper would benefit from a more quantitative assessment of the extent to which meQTLs influence clock portability.

We agree that the meQTL likely influence the clocks in different ways and that the ascertainment of the meQTLs in different populations makes direct comparisons challenging. To more directly link meQTL to clock performance, we identified 56 Horvath clock CpG sites whose methylation levels significantly associate with increased clock error in the MAGENTA study individuals. Of these, 42 (75%) are affected by an meQTL, including nine that are affected by an African ancestry-differentiated meQTL. As such, meQTL, and specifically meQTL that were likely not present in the training data of the Horvath clock, associated with both the methylation of CpG sites and clock error. However, as the reviewer suggests, determining causality among these factors is challenging. Given our incomplete knowledge of meQTL in different ancestries, we have added caveats to our conclusions about the effect of meQTL on clock portability.

The paper implies that methylation clocks have an inferior ability to predict AD risk in admixed populations relative to white individuals, but the difference between white AD patients and controls is not significant when correcting for multiple testing. This nuance should be made more explicit.

We agree that the signal is not strong in the white cohort; however, it is similar in magnitude to previous studies. As outlined in response to Reviewer 1’s Point 5, we have now added power calculations that indicate reasonable power (≥72%) to detect small effect sizes (0.5 year increase) in the white, Puerto Rican and African American cohorts. We now interpret the AD association tests in the context of these power calculations and multiple testing correction.

Finally, this paper overlooks the possibility that environmental exposures co-vary with genetic ancestry and play a role in decreasing the accuracy of methylation clocks in genetically admixed individuals. Quantifying the impact of environmental factors is almost certainly outside of the scope of this paper. However, it is worth acknowledging the role of environmental factors to provide the field with a more comprehensive overview of factors influencing methylation clock portability. It is also essential to avoid the assumption that correlations with genetic ancestry necessarily arise from genetic causes.

We entirely agree and have now clarified the scope of our analyses and importance of environmental factors in the revision. We intersected clock CpGs with enviromental-factor-associated CpGs from multiple epigenome-wide association studies (EWAS) and found overlaps that suggest an environemtnal contribution to differences in clock CpG methylation. However, given the lack of environmental data on the MAGENTA study individuals, as well as the lack of datasets for replication, we cannnot directly compare the environmental and genetic contributions to clock accuracy. Nevertheless, the new analyses in the revision highlight the contribution of both genetic and environmental factors to lack of portability for certain methylation clocks.

**Reviewer #2 (Recommendations for the authors):**

(1) Line 64: An association between methylation patterns and genetic ancestry does not presuppose that meQTLs vary in frequency between genetic ancestries; environmental factors could also play a role. It would be nice to comment on this further in the Introduction.

We agree that environmental factors likely play a role in the decrease in methylation clock performance in admixed populations. We have added text highlighting this in the revised Discussion. Regarding meQTL, we agree that associations between methylation patterns and genetic ancestry do not necessarily imply that meQTL will vary in frequency between genetic ancestries. However, our new analyses in the revision find African-ancestry differentiated meQTL that associate with Horvath clock CpG methylation levels and overall clock error (Figure 6E-F and Supplementary Figure 13).

(2) Line 116 implies Puerto Ricans have “substantial amounts of African ancestry” but the median ancestry is 15% (which is not much more than the Peruvian and Cuban cohorts).

Thank you for pointing this out. We have clarified this statement in the text. While the median proportion of African ancestry in Puerto Ricans is 15% (vs. 6% and 2% for the Peruvian and Cuban individuals in MAGENTA), there are many individuals with substantially higher African ancestry. The upper quartile is >25% and several Puerto Ricans have >50% African ancestry.

(3) In Figure 2B, Puerto Ricans have worse accuracy than Peruvians but a higher proportion of inferred CEU ancestry, which is interesting and defies intuition - is there any hypothesis for why this might be the case?

In light of our new meQTL analyses, we hypothesize that the African ancestry differentiated meQTL that affect Horvath clock CpGs drive the increase in clock error for these individuals, despite having more European ancestry across their genome. Given that the Peruvians (and Cubans, for that matter) hold very little African ancestry, and also very few of the African-differentiated meQTL, this could explain some of the large difference in clock errors for the cohorts.

(4) Figure 2C would be improved with confidence intervals.

We thank the reviewer for this suggestion and have added confidence intervals for Figure 2C.

(5) It’s interesting that the correlation with Cubans is positive in Figure 3B (for one clock, significantly so). Is there any rationale for this?

We noticed this as well, but have not been able to come to a definitive conclusion. It is possible that environmental factors contribute. However, the Cuban cohort is the smallest in MAGENTA (22 cases and 21 controls) and the none of the differences are statistically significant, so more investigation in a large cohort is required.

(6) Line 231: Which population(s) is allele frequency estimated in?

This is the global frequency reported in gnomAD, which is calculated across all populations in gnomAD v3.0. As noted above, we now also report allele frequencies by gnomAD population (Supplementary Figure 9).

(7) Were the meQTLs pruned? How many independent variants are there per methylation site? It would be nice to see a distribution for the sites in the Horvath clock.

We now report the distribution of meQTL across clock CpG sites. The mean number of variants is 108; the median is 36; and the maximum is 1,699. We have now included a plot of the distribution for all 271 (out of 353) Horvath clock CpG sites (Supplementary Figure 14). We did not perform any pruning in these initial results for several reasons. First, we sought to demonstrate the great potential for meQTL to influence these CpGs and to compare the distributions of these common meQTL across populations (based on gnomAD data). Second, identifying the causal variant or variants is challenging. Given that many of these meQTLs likely reflect redundant signals, for the new analyses of African-differentiated meQTL, we restrict to a single variant per clock CpG site. We focus on the variant with the greatest absolute beta, as reported by the original meQTL study from which the variant originates.

(8) Figure 5C might benefit from a geom density rather than overlapping bar plots; the trends are hard to see.

We appreciate the reviwer’s suggestion and have now reworked the figure and based it on just the density curves so that readers may better appreciate the differences in allele frequencies.

(9) Several figures would be more legible with larger font sizes.

We appreciate this recommendations and have made the font sizes for all plots larger and more legible.

**Reviewer #3 (Public review):**

This manuscript examines the accuracy of DNA methylation-based epigenetic clocks across multiple cohorts of varying genetic ancestry. The authors find that clocks were generally less accurate at predicting age in cohorts with large proportions of non-European (especially African) ancestry, compared to cohorts with high European ancestry proportions. They suggest that some of this effect might be explained by meQTLs that occur near CpG sites included in clocks, because these variants may be at higher frequencies (or at least different frequencies) in cohorts with high proportions of non-European ancestry relative to the training set. They also provide discussions of potential paths forward to alleviate bias and improve portability for future clock algorithms.

The topic is timely due to the increasing popularity of DNA methylation-based clocks and the acknowledgment that many algorithms (e.g., polygenic risk scores) lack portability when applied to cohorts that substantially differ in ancestry or other characteristics from the training set. This has been discussed to some degree for DNA methylationbased clocks, but could of course use more discussion and empirical attention which the authors nicely provide using an impressive and diverse collection of data.

Thank you for this summary.

The manuscript is clear and well-written, however, some key background was missing (e.g., what we know already about the ancestry composition of clock training sets) and most importantly several analyses would benefit from being taken one step further. For example, the main argument of the paper is that ancestry impacts clock predictions, but this is determined by subsetting the data by recruitment cohort rather than analyzing ancestry as a continuous variable. Extending some of the analyses could really help the authors nail down their hypothesized sources of lack of portability, which is critical for making recommendations to the community and understanding the best paths forward.

Thank you for this suggestion. As noted in our response to Reviewer 2’s Point 1, we have analyzed ancestry as a continuous variable and found that the proportion of African ancestry in the genomes of the MAGENTA individuals significantly associates with increased difference in chronological and predicted age, even after controlling for chronological age (1.46 years more error for 100% vs. 0% African ancestry; p = 0.039). As outlined below, we have also added details on the training of previous clocks and the important additional previous work highlighted by the Reviewer.

**Reviewer #3 (Recommendations for the authors):**

Major comments

There is previous literature addressing who is in the training set for methylation clocks. To my knowledge, this work has been primarily led by Nancy Krieger. It would be a valuable addition to discuss her work (and any similar work by other investigations) in the introduction. In other words, what do we currently know about the degree of bias in the training sets for methylation-based clocks? The assumption of the introduction is that the training sets are overwhelmingly European ancestry (which I assume is true) but I think some quantitative information about this would be helpful for understanding the source and magnitude of the problem.

We thank the reviewer for bringing the work of Dr. Nancy Krieger to our attention. It directly supports the rationale for this study: the sociodemographic characteristics of the individuals used to train these clocks are poorly reported, limited to outdated population descriptors (for example, the use of “Caucasians” to describe some of the individuals used to train the Horvath and the Hannum clocks) or race and ethnicity labels. Moreover, where labels are available for training individuals, they tend to underrepresent the individuals of diverse backgrounds, as in the Horvath clock. We have incorporated Dr. Krieger’s work into the Introduction, including details of how this supports the rationale and purpose of our study.

Related to the above comment, there has been pretty extensive previous work on the effects of race and ethnicity on epigenetic clock estimates (e.g., https://genomebiology.biomedcentral.com/articles/10.1186/s13059-016-1030-0), and that seems like it could be more explicitly weaved into the introduction and discussion.

We thank the reviewer for highlighting this relevant article. We have added discussion of it into the Introduction. Several factors make direct comparison with our results challenging. First, the grouping of individuals based on race and ethnicity without consideration of genetic ancestry complicates comparisons. Race and ethnicity commonly do not match genetic ancestry components (see Gouveia et al., 2025 https://www.cell.com/ajhg/fulltext/S00029297(25)00173-9). Second, the study reports differences in epigenetic age accelerations (intrinsic and extrinsic) in individuals from various race and ethnic groups. It does not directly evaluate the accuracy of the epigenetic age predictions in these groups. Thus, it is challenging to interpret whether the differences in acceleration are driven by biological factors or biases in the performance of the clocks themselves.

The main analysis that felt like it was missing was asking whether the age deviations are larger for individuals with greater proportions of African ancestry. The authors have the ability to analyze ancestry as a continuous variable, but instead performed analyses in various a priori subsets of the data; the subsets do have average differences in ancestry, but also there is heterogeneity within groups. Given that the authors calculated admixture proportions already, it seems like a missed opportunity not to use these estimates. This would also sidestep the issue of the problematic labels applied to the subsets, which mix ancestry, nationality, and race terms (note that I thought the legacy reasons why these labels are used were well-explained, but they are nevertheless problematic for biological explanations that center on ancestry/genetic information as the driver of bias).

We appreciate the reviewer’s suggestion to investigate clock accuracy in the context of African ancestry proportions. As noted in the response to Reviewer 2’s Point 1, we modeled the clock error as a function of the fraction of African ancestry of each individual, adjusting for an individual’s chronological age. The proportion of African ancestry is significantly associated with increased Horvath clock error (p = 0.039), with the clock estimated to give less accurate age predictions by 1.46 years for individuals with 100% African ancestry compared to no African ancestry. We now report this in the Results.

Another missed analysis opportunity occurs in lines 259-261, where the authors state “Thus, the clock with the largest decrease in performance in admixed cohorts (in terms of predicting chronological age and identifying age acceleration in AD) has the most and largest fraction of meQTLs influencing its CpGs.” This is another place where the authors make generalizations about a given cohort based on average ancestry rather than testing the claim empirically on an individual basis (e.g., by examining the number of meQTL variants a given individual is heterozygous for or has the non-European allele for).

We thank the reviewer for this comment. This feedback motivated us to evaluate the relationship between differences in meQTL frequencies and methylation clock error. We found differences in meQTL frequency in the MAGENTA individuals, specifically many of the clock CpG affecting meQTL are most common in the African American cohort, consistent with our theory (Figure 6E,F). Nonetheless, there are 84 Horvath clock CpGs (24%) that are differentially methylated in AFR individuals, and 56 of these are affected by an meQTL, including 11 that are affected by an African ancestry-differentiated meQTL (Figure 6G). Finally, we find that 42 Horvath clock CpG sites in MAGENTA individuals with methylation levels that are significantly associated with increased clock error, and that are also affected by an meQTL (Figure 6B). However, at the individual level we do not find a clear relationship between the number of meQTL or ancestry-differentiated meQTL and methylation clock error. In light of these data, we have reframed our conclusions to state that meQTL likely contribute to clock error, while also being clear that they are not the sole cause.

Can the authors explain or offer an investigation into why predicted age is often better in Cubans than Whites? They gave much attention to the opposite effect (of similar magnitude) in African Americans and Puerto Ricans but didn’t really discuss the surprisingly accurate prediction in Cubans.

We did not focus on the results in the Cuban cohorts for several reasons. As discussed in response to Reviewer 2’s comment, the Cuban cohort had the smallest sample size (22 cases and 21 controls). Thus, while the correlation between methylation age and chronological age is similar to Whites, and in a few cases higher, the differences were not statistically significant. Second, looking at other error metrics, like mean absolute error, the clocks are comparatively less accurate in Cubans than on the White cohort (Supplementary Table 2). Finally, the clocks consistently find that Cubans with AD have lower predicted age than controls, though this is only significant for the ZhangEN clock. However, given these inconsisencies and the very small sample size, we caution against over-interpretation of these results. We clarify this in the manuscript and suggest that more work is needed on larger Cuban cohorts before any clear conclusions can be made.

I was not a conceptual fan of the ensemble clock. The clocks are trained on very different things (e.g., chronological age versus clinical biomarkers) and are designed to capture different aspects of biology. Without more validation and motivation, I don’t think it makes sense to average values that are not designed to measure the same thing.

We agree that combining the first and second-generation clocks for the task of age prediction is not sensible. However, for AD risk stratification, combining values from multiple clocks that capture different aspects of biology and aging could be beneficial. As mentioned in the main text, we took inspiration from approaches in polygenic risk scores, as well as the broader machine learning field, where ensembling often makes for better predictors. Nonetheless, consistent with the Reviewer’s intuition, we do not see improvement here.

Minor comments

(1) Typo in line 91.

Thank you for bringing this to our attention. Fixed.

(2) Lines 111-115, sample sizes would be helpful.

We have added the sample sizes of the non-demented controls that were used to calculate these correlations in each cohort.

(3) Line 137-138, the correlation stats would be helpful here. This is a common issue throughout the paper, more in-text statistics would help readers to evaluate the authors’ claims. For example, lines 249-251 as well. The authors refer the reader to Figure 5C, which itself has no statistics, this has two plots so it’s unclear which the authors are putting forward as the primary evidence.

We have added more statistical details in the text and figures to address this comment. In this instance, we have removed the referenced figure.

(4) Lines 258 and 261, I believe the authors report the same result in both these lines.

Thank you for pointing out this lack of clarity. These lines report different, but related, results about the frequency of clock-affecting meQTL in different ancestral contexts. The first reports the frequency of clock CpGaffecting meQTL in individuals of African ancestry across all of gnomAD. The second result gives the frequency of those meQTL in different local ancestry backgrounds in admixed individuals. This is distinction is relevant since admixed individuals’ genomes are mosaics of multiple genetic ancestries. As such, a genetic variant might be present in haplotype whose ancestry is not in line with expectations based on global ancestry (e.g., an African American individual inherits a genetic variant within a European ancestry block). This local ancestry difference could modify the effect of the variant or obscure causal variants. Given the potential for confusion and similar results considering global and local ancestry context in this case, we have focused on the first result in the Main Text.

(5) Somewhere, it would be helpful to provide the distribution/range of ages broken by cohort. Similarly, I didn’t see the breakdown of AD versus control cases within each cohort. Both of these features will impact power within a given cohort for certain analyses.

We have added the distribution of ages by cohort in Supplementary Figure 1. Table 1 provides a breakdown of cases versus controls for each of the cohorts in the MAGENTA study.

(6) Figure 3 is pretty hard to read. It would also be helpful if the authors put the white cohort in Figure 3A as a ’baseline’ comparison, as they use this as the baseline comparison in the text.

We have made these changes to the figure and used larger text overall.

(7) The various acronyms in the labels in Figure 5 are not explained. For Figure 5C - this is over-plotted and therefore hard to see.

We have added the full population descriptors from gnomAD to the boxplots showing allele frequencies (Figure 6E). In addition, what used to be Figure 5C has been simplified and moved to Supplementary Figure 12.

(8) The authors correct for cell type heterogeneity, which is known to vary across populations and can impact clock estimates. However, as far as I can tell, the cell type proportion estimates are coming from the DNA methylation data. The deconvolution algorithms for cell type proportions also have the same problem as the clocks of being trained on a very specific subset of human genetic and environmental diversity. Do the authors have any empirically derived estimates of cell type heterogeneity to sanity-check these deconvolution estimates? At the very least, it would be helpful to acknowledge this limitation.

We thank the reviewer for commenting on this. There are no empirically derived estimates of cell type counts for the samples in the MAGENTA study. This is an inherent limitation of our study, and we have included text to make note of this.

(9) There are very different sample sizes for each group, did the authors consider that their null results for the AD analyses in different cohorts are just a lack of power? This could be evaluated with power analyses or by comparing against sample sizes from similar studies in the literature.

We agree that this is an important analysis and have added it to the manuscript. Given these small effect sizes, accounting for statistical power is essential for interpretation of our results. We performed power calculations based on an effect of the size observed in previous studies (0.5 year acceleration). Considering the full study, we have 86% power to detect an effect of this size. Stratifying by cohorts, we have 75% power for the African Americans, 72% for the Puerto Ricans, 72% for the Whites, 65% for the Peruvians, and 47% for the Cubans. Thus, we have high enough power that the consistent lack of association observed outside of the White cohort in MAGENTA is likely meaningful. Based on these calculations, there is only a 1% chance that we would not observe an effect in any of the other cohorts if the effect was present across cohorts. Nonetheless, we have added caveats about power and the small sample size to our suggestion that the reduced accuracy of the clocks contributes to the lack of association outside of Whites.

(10) There has been a fair amount of discussion recently that single CpG-based clocks are much more variable than clocks that combine information across CpG sites, either using PC-based or window-based approaches. For example, the PC clock R package from the Levine Lab (https://github.com/MorganLevineLab/PC-Clocks) is very easily implemented and generally gives much less variable age estimations than site-level clocks. It would be nice to consider integrating or discussing these later-generation clocks as ways to improve clock performance in diverse human groups.

We thank the reviewer for their suggestion to apply the principal component clock to account for potential technical variation. As outlined in the new section “Principal component versions of the methylation clocks also have lower age prediction accuracy for genetically admixed individuals,” using the principal component version of the Horvath clock did not result in consistent improvement in age prediction accuracy or generalization across MAGENTA cohorts (Supplementary Figures 4 and 5). The lower accuracy for age prediction in individuals with substantial African ancestry were present for the PC clock in the replication cohorts, just as in the MAGENTA cohorts (Supplementary Figure 6)
